# Infection With *Clostridioides difficile* Attenuated Collagen-Induced Arthritis in Mice and Involved Mesenteric T_reg_ and T_h2_ Polarization

**DOI:** 10.3389/fimmu.2020.571049

**Published:** 2020-10-30

**Authors:** Christian Johann Schmidt, Katharina Wenndorf, Meinolf Ebbers, Johann Volzke, Michael Müller, Julia Strübing, Katja Kriebel, Susanne Kneitz, Bernd Kreikemeyer, Brigitte Müller-Hilke

**Affiliations:** ^1^ Laboratory for Clinical Immunology, Core Facility for Cell Sorting & Cell Analysis, University Medical Center Rostock, Rostock, Germany; ^2^ Department of Tropical Medicine and Infectious Diseases, University Medical Center Rostock, Rostock, Germany; ^3^ Microbiology, Institute for Life Sciences, University of Rostock, Rostock, Germany; ^4^ Physiological Chemistry, Theodor Boveri Institute (Biocenter), University of Wuerzburg, Wuerzburg, Germany; ^5^ Institute of Medical Microbiology, Virology and Hygiene, University Medical Center Rostock, Rostock, Germany

**Keywords:** ****rheumatoid arthritis, collagen-induced arthritis, *Clostridioides difficile*, microbiome, T_reg_-polarization

## Abstract

**Objectives:**

Rheumatoid arthritis is an autoimmune disease with multifactorial etiopathogenesis. Among the environmental factors, mucosal infections and the inducing pathobionts are gaining increasing attention. We here set out to explore the gut-joint-axis and the impact of *Clostridioides difficile* infection on subsequent arthritis.

**Methods:**

We combined *C. difficile* infection in DBA/1J × B10.Q F1 mice with collagen induced arthritis (CIA). Mice were infected *via* oral gavage and infection was monitored by weight loss, colonic histology, and antibodies against bacteria. Scoring of arthritis was performed macroscopically. Intestinal microbiomes were analyzed and immune responses were monitored *via* quantification of transcription factor-specific mRNA isolated from the inguinal and mesenteric lymph nodes.

**Results:**

Infection with *C. difficile* VPI 10463 resulted in significant weight loss and severe colitis yet accelerated the reversal towards the original microbiome after antibiotic treatment. Spontaneous clearance of VPI 10463 infection reduced the incidence of subsequent CIA and led to mesenteric T_reg_ and T_h2_ polarization. However, this attenuating effect was abrogated if VPI 10463 was eradicated *via* vancomycin followed by fecal microbiota transplantation. Moreover, VPI 10463 infection following the onset of CIA lacked therapeutic potential.

**Conclusion:**

Our results demonstrate that infection with *C. difficile* VPI10463 induced an inflammation of the gut that protected from subsequent arthritis development in mice. Both, microbial changes to the gut and immune cell mobilization and/or polarization may have contributed to arthritis protection. The prospect of potential therapeutic benefits resulting from *C. difficile* infections or some byproduct thereof call for further experiments that help elucidate exact mechanisms.

## Key Messages

- Mucosal infections and their inducing pathobionts are increasingly implicated in the pathogenesis of rheumatoid arthritis
- Spontaneous clearance of *Clostridioides difficile* infection reduced the incidence of subsequent CIA
- Spontaneous clearance of *Clostridioides difficile* infection led to mesenteric T_reg_ and T_h2_ polarization

## Introduction

Rheumatoid arthritis (RA) is one of the most common autoimmune diseases and primarily affects the joints, even though it can develop into a systemic disease with various extra-articular manifestations (1). In the absence of adequate therapy, chronic joint inflammation leads to pain, dysfunction, and disability (2). With a worldwide prevalence of about 1% and an annual economic burden of at least 45 billion € in Europe, RA is also of high socioeconomic relevance (3). And even though the last decades witnessed a marked improvement of early diagnosis and the development of numerous efficient drugs, there is neither a cure nor has the pathogenesis of RA been fully unraveled.

RA is a multifactorial disease and the genetic factors contributing to disease occurrence were estimated to range between 53 and 65% (4). This in turn means that at least one third of RA susceptibility is due to environmental variation. Among these, sex and age play a role as do lifestyle, diet, and infection (4–6). The quest for an infectious micro-organism that triggers the pathogenicity of RA has been the target of considerable study however, the absence of a clustering of the disease in time or place and the likelihood of a delay between infection and disease onset have hampered conclusive evidence. Moreover, the many clinical facets of RA, the diversity of responses to targeted therapies as well as the broad range of genes associated with it leave space for more than one infectious agent as a trigger (7).

Among the early micro-organisms that were suspected to play a role in RA pathogenesis were Epstein-Barr and human parvovirus (8). More recently, bacteria like *Proteus mirabilis, Escheria coli* ssp., *Klebsiella pneumonia*, *and Porphyromonas gingivalis* have gained increasing interest (9–11). These bacteria induce mucosal immune reactions in the urinary tract, the gut, the lung, and the gingiva, respectively. The etiological models involved imply tissue stress at a site distant from the joints, the generation of neo-autoantigens, loss of tolerance by molecular mimicry, and bystander activation of the immune system (5, 12). As not only diet but also dysbiosis of the gut and subsequent deviation of the immune response have frequently been linked to RA, the mucosal microbiome has increasingly gained attention with respect to disease induction (13–15).

One of the most common reasons for dysbiosis of the gut is the administration of antibiotics, with the global antibiotic consumption having increased by 65% between 2000 and 2015 (16). This increase combined with classic risk factors like age, diseases of the intestines, prolonged hospital stays and immunosuppression led to a rise in *Clostridioides difficile* infections (17). *C. difficile* produces two major virulence factors, enterotoxin A and cytotoxin B, that induce cytotoxic damage to the intestinal cells followed by diarrhea and inflammation of the intestines. Indeed, *C. difficile* is considered the most important pathogen for nosocomial diarrhea and its clinical manifestations range from mild self-limiting diarrhea to fulminant colitis, accompanied by complications such as toxic mega colon, bowel perforation, sepsis, and death (18, 19).

In the present study we raised the question if *C. difficile* infection and subsequent dysbiosis and colitis would impact the pathogenesis of arthritis. To address this question we turned to a mouse model because not only are the microbiomes of mice and man largely similar, but collagen induced arthritis (CIA) in the mouse is a widely used model for RA (20). In a first approach we tested three differently human-pathogenic *C. difficile* strains for their pathogenicity in the DBA/1J x B10.Q F1 mouse. Thereafter we induced CIA, scored for its incidence and severity and monitored the respective changes to the gut microbiome. Our results indicate that—contrary to our expectations—*C. difficile* infection exerted attenuating effects on subsequent arthritis.

## Materials and Methods

### Mice

DBA/1J mice were originally purchased from Charles River (Sulzfeld, Germany) and B10.Q from Jackson Laboratory. The F1 generation resulting from DBA/1J x B10.Q mating was generated in our animal care facility under specific pathogen free conditions. They were housed in cages of 1–5 mice in a climate-controlled environment with a 12 h light/dark cycle with food (ssniff, Soest, Germany) and water given *ad libitum*. After being marked and acclimatized, 8 to 12 week old mice (21) were entered into the experiments and subsequently weighed every other and every third day respectively, depending on the phase of experiment. At endpoint, mice were euthanized *via* cervical dislocation under deep anesthesia with Esketamine (0.75 mg/10 g of body weight, bela-pharm, Vechta, Germany) and Xylazine (0.05 mg/10 g of body weight, Bayer AG, Leverkusen, Germany) administered by intraperitoneal injections. All experiments were approved by the local state’s animal care committee (LALLF M-V) 7221.3-1.1-035-16 and 7221.3-1.1-039-18 and were performed according to the guidelines for animal experiments.

### Bacterial Strains, Infection, and Eradication

*Clostridioides difficile* strains 630, 2K14, and VPI 10463 were kindly provided by Nigel P. Minton (University of Nottingham, Centre for Biomolecular Sciences, Nottingham, UK). All strains were grown under anaerobic atmosphere (N_2_ with max. 5% H_2_) using Brain-Heart-Infusion Medium (HiMedia Laboratories GmbH, Einhausen, Germany) supplemented with 5 g/l yeast extract (Biolab Zrt. Budapest, Hungary) and 0.1% L-cystein (Acros Organics part of Thermo Fisher Scientific, Waltham, MA, USA). *C. difficile* strains 630 and 2K14 were grown until they reached mid-logarithmic phase. The time point for cell harvest was variable according to medium- and growth condition-specific growth rates μ [h−1] and doubling times td [min]. Bacterial cultures were centrifuged, washed with PBS, and resuspended in glycerin/BHI medium (1:10). Aliquots were kept frozen at −80°C until immediately before the respective experiments. For inoculation, aliquots were thawed, vortexed, and then diluted to a concentration of 1x10^5^ cfu per 200 µl with DPBS (Thermo Fischer Scientific, Waltham, MA, USA). Due to a high loss of cfu during freezing and thawing *C. difficile* VPI 10463, this strain was freshly prepared for each experiment. *C. difficile* VPI 10463 was cultivated for 24 h, adjusted to an optical density of 1.5 in BHIS, and aliquoted into 1 ml aliquots. For cfu counts of all strains, 100 µl were used to perform serial dilutions onto BHIS agar plates. The plates were incubated for another 24 h and the cfu/ml were calculated.

#### Pretreatment

For the pretreatment we followed a protocol as described previously (21). Shortly, an antibiotic mixture of 0.4 mg/mL kanamycin sulfate (Carl Roth, Karlsruhe, Germany), 0.035 mg/mL gentamicin (Gentamicin 40 mg/ml, ratiopharm, Ulm, Germany), 850 U/m colistin (ColistiFlex powder 1x10^6^ U/phial, InfectoPharm, Heppenheim, Germany), 0.215 mg/mL metronidazole (metronidazole 5 mg/ml, Braun Melsungen AG, Melsungen, Germany) and 0.045 mg/mL vancomycin (vancomycin 5 mg powder/bottle, Eberth, Ursensollen, Germany) in sterile water was prepared daily. This corresponded to the approximate daily dose used for each antibiotic such as kanamycin (40 mg/kg), gentamicin (3.5 mg/kg), colistin (4.2 mg/kg), metronidazole (21.5 mg/kg), and vancomycin (4.5 mg/kg). The concentrations of antibiotics in the water were calculated based on the average weight and expected water consumption of the mice. Mice were pretreated with the antibiotic mixture in drinking water for 3 days, followed by a 2 days washout and a single intraperitoneal injection of 10 mg/kg clindamycin (clindamycin 600 mg/4 ml phiol, MIP, Blieskastel-Niederwürzbach, Germany) on day 6. Another day later, mice received 200 µl either fresh *C. difficile* VPI 10463 in bacterial medium or thawed *C. difficile* 630 or 2K14 in glycerin and PBS *via* oral gavage (Feeding Needle, 20G, Fine Science Tools, Heidelberg, Germany).

**Infections** with *C. difficile* were performed under a laminar flow hood using appropriate personal protective equipment. For handling of the mice, work surfaces were sterilized using sporicidal disinfectant Dismozon plus (BODE Chemie GmbH, Hamburg, Germany) in order to prevent cross-contamination of spores between experimental groups.

#### Scoring and Treatment of *Clostridioides difficile* Infections

After infection, mice were scored at least once a day for body weight, diarrhea, general condition, and behavior. Weight loss turned out to best reflect diarrhea and was chosen to monitor severity of *C. difficile* infections (CDI). Loss of more than 5% of the original body weight led to an oral analgetic and antispasmic therapy with metamizole (novaminsulfon 500 mg/ml, Lichtenstein/Zentiva, Frankfurt a.M., Germany) in the drinking water. Mice losing 20% of their initial weight were monitored twice in 24 h and if symptoms did not ameliorate within this time were euthanized.

#### Eradication of *Clostridioides difficile* Infections and Fecal Microbial Transplant

Eradication of *C. difficile* adhered to a previously described protocol and included vancomycin (20 mg/kg) in the drinking water for 5 days **(** 22). Thereafter, mice received a fecal microbial transplant (FMT) to prevent a relapse after vancomycin treatment. To that extent, eight fecal pellets collected from two conventionally kept and untreated mice were suspended in 1.5 ml PBS, vortexed, centrifuged and 100 µl of supernatant were fed to each *C. diff.* infected mouse *via* oral gavage (23, 24).

### Collagen-Induced Arthritis

The induction of arthritis (CIA) was performed as described previously (25) with slight modifications. Briefly, for primary immunization on experimental day 28 (or day 1, respectively), mice received 140 mg bovine type II collagen (MD Bioscience, Egg b. Zürich, Switzerland) in 0.1 M acetic acid emulsified in an equal quantity of complete Freund’s adjuvant (CFA, Becton, Dickinson and Company, Franklin Lakes, NJ, USA). Three weeks later, on experimental day 49 (and 22, respectively), mice were boosted with 140 mg bovine type II collagen in 0.1 M acetic acid emulsified in an equal amount of incomplete Freund’s adjuvant (IFA). For both procedures, mice were placed in restrainer and injected subcutaneously with 70 µl at each side of the tail base. Every third day after the boost, mice were scored for macroscopic signs of arthritis, such as swelling and/or erythema on hind and fore limbs. To that extent, each affected tarsal, metatarsal, carpal, or metacarpal joint was rated with five points and each affected digit with one point, whereby a maximum score of 15 points per paw or 60 points per mouse could be reached in total. At the end of the experiments, all paws were removed and fixed in formalin 4% for 2 weeks, washed, decalcified, and then stored in 70% alcohol for later histological analyses.

### Histology of Intestines

Upon euthanasia, intestinal tissues were removed, cleaned with PBS, and fixed in a Swiss roll (26) in 4% formalin for 2 days and then placed in 70% ethanol before being embedded into paraffin and stained with hematoxylin and eosin (H&E). Images from 4 µm thin tissue sections were taken by standard light microscop (Axioskop 40 Mikroskop Carl Zeiss AG, Oberkochen, Germany) camera (AxioCam MRc 5 Carl Zeiss AG, Oberkochen, Germany).

### Serum Analysis

At the end of the experiments, about 1.5 ml of blood per mouse were obtained from the orbital vein plexus under deep anesthesia before cervical dislocation. The serum was extracted and stored at −20°C for further processing.

#### *Clostridioides difficile* Antibodies

Antibody titers against *C. difficile* toxin A and B were analyzed by using *C. difficile* anti-TcdA/B ELISA for mouse (TGC Biomics, Bingen, Germany). In short, mouse serum was applied at a dilution of 1:200 on plates coated with either toxin A or toxin B and incubated for 1 h at 37°C. After washing, the plates were incubated with 100 ml of peroxidase conjugated anti-mouse antibody for another 30 min. Finally, color reaction was performed adding 100 ml TMB-substrate and measurements of the extinction were done with an automated plate reader (HT3, Anthos Mikrosysteme GmbH, Krefeld, Germany) at 450 nm.

#### Collagen Antibody

Antibody titers against collagen type II were analyzed by coating Nunc MediSorp ELISA plates (Thermo Fisher Scientific, Waltham, MA, USA) with bovine (MD Bioscience, St. Paul, MN, USA) and murine (Chondrex Inc., Redmond, WA, USA) collagen type II at 20 μg/ml each in carbonate/bicarbonate buffer overnight at 4°C. Sera were applied at a dilution of 1:6,000 for 1.5 h at RT. After washing, the plates were incubated with diluted rabbit anti-mouse IgG for 1 h at RT. Thereafter, bound antibodies were detected as described for *C. difficile* IgG detection.

### Gut Microbiota Analysis

#### Fecal DNA Extraction

Fresh stool samples were collected on days 1, 7, 9, 28 and at endpoint and stored at −80°C until use. One sample each was collected from all animals however, samples from one cage were pooled and numbers of pellets pooled depended on the number of mice that cohoused in one cage. The CDI group (n=8) thus resulted in five stool pellets per time point, the CDI+CIA group (n=7) resulted in four stool pellets per time point, the medium group (n=6) in three stool pellets, and the CIA group (n=6) in two stool pellets. For further analysis, stool was thawed, homogenized *via* Fastprep-24 (MP Biomedicals, Santa Ana, CA, USA) with 6 m/s for 1 min using the ZR-96 BashingBead Lysis Tubes (Zymo Research, Irvine, CA, USA) and DNA was eluted using the ZymoBIOMICS DNA Miniprep Kit (Zymo Research, Irvine, CA, USA) following the manufacturer’s instructions. DNA concentration and integrity were measured *via* NanoDrop 2000 spectrophotometer (Thermo Fisher Scientific, Waltham, MA, USA).

#### 16SrRNA PCR

Amplicon PCR was performed with microbial genomic DNA using a concentration of 5 ng/µl in 10 mM Tris pH 8.5. PCR amplification of the V3/V4 regions of bacterial 16S rRNA encoding gene was carried out using the primers Pro341-XT (TCG-TCG-GCA-GCG-TCA-GAT-GTG-TAT-AAG-AGA-CAG-CCT-ACG-GGN-BGC-ASC-AG) and Pro805-XT (GTC-TCG-TGG-GCT-CGG-AGA-TGT-GTA-TAA-GAG-ACA-GGA-CTA-CNV-GGG-TAT-CTA-ATC-C) which resulted in amplicon sizes smaller than 550 bp. The details of library constructions, such as index PCR, PCR clean-up 2, library quantification, normalization, and pooling were performed corresponding to the Illumina “16S Metagenomic Sequencing Library Preparation” protocol.

Bioanalyzer DNA 1000 chips (Agilent Technologies, Santa Clara, CA, USA) and Qubit kits (Thermo Fischer Scientific, Waltham, MA, USA) were applied for quantity and quality controls of each individual sample library and the final library pool; 5 pM of the final library mixture, containing at least 5% PhiX control, was exposed to one individual sequencing run using a 2x250 or 2x300 cycle 3 reagent cartridge on an Illumina MiSeq machine. All raw data fastq files were used for sequence data analyses.

#### Sequence Data Analysis

Quality filtering (permitted length: 440–466 bp, no ambiguous bases allowed), merging of duplicated sequences and alignment to the reference database (https://www.mothur.org/wiki/Silva_reference_files#Release_128) were done using mothur (27). Only OTUs with total abundance >=3 were considered. Sequences from archaea, chloroplasts eukaryote, and mitochondria were removed. For descriptive community analysis and PCA plots, the CRAN package ‘vegan’ has been used (https://cran.r-project.org/web/packages/vegan/index.html). Similarity was calculated as Jaccard index, as a measure for dissimilarity Bray-Curtis has been used. For statistical analysis of differences between read counts at different time points, Kruskal-Wallis tests with pairwise multiple comparisons by Nemenyi-tests were used. The raw sequencing fastq files were submitted to and are available at the BioProject database under the ID “PRJNA615786.”

### Quantitative Real Time PCR

Inguinal and mesenteric lymph nodes were harvested, stored overnight at 4°C in 200 µl RNAlater (Thermo Fisher Scientific, Waltham, MA, USA), and thereafter stored at −80°C until further processing. For RNA preparation, specimen were thawed on ice, then transferred in FastPrep lysing matrix A (MP Biomedicals Germany GmbH, Eschwege, Germany) and 600 µl buffer RLT (Qiagen, Hilden, Germany). Tissues were homogenized with FastPrep (MP Biomedicals Germany GmbH, Eschwege, Germany) at 6.0 m/s for 45 s. Homogenates were then centrifuged at 16,000 x g for 1 min using a Microfuge 16 (Beckman Coulter, Brea, CA, USA). Proteins were extracted from the pellets by adding 600 µl Acid-Phenol : Chloroform pH 4.5 (Thermo Fisher Scientific, Waltham, MA, USA), manual mixing for 30 s and then centrifuging at 16,000 x g for 5 min at RT. RNA was extracted from the aqueous phase *via* RNeasy Mini Kits (Qiagen, Hilden, Germany). The RNA contents were measured using NanoDrop (Thermo Fisher Scientific, Waltham, MA, USA) and complementary DNA (cDNA) was synthesized by performing real-time PCR according to High-Capacity cDNA Reverse Transcription Kit Users Guide (Thermo Fisher Scientific, Waltham, MA, USA) using Mastercycler gradient (Eppendorf AG, Hamburg, Germany). Quantitative PCR was performed using ABI 7900 HT Fast Real Time PCR (Applied Biosystems, Waltham, MA, USA) according to the TaqMan Gene Expression Master Mix User Guide (Applied Biosystems, Waltham, MA, USA). The following TaqMan gene expression assays were used: Gapdh (Mm99999915_g1), Tbx21 (Mm00450960_m1), Gata3 (Mm00484683_m1), Foxp3 (Mm00475162_m1), and Rorc (Mm01261022_m1).

### Statistical Analyses

**Figure 1:**
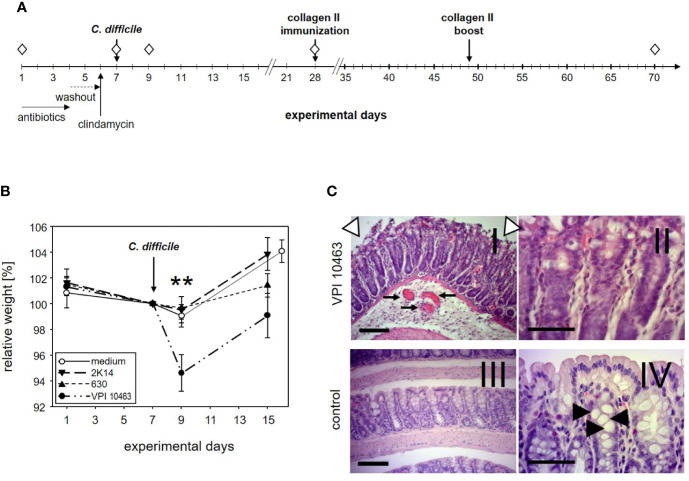
DBA/1J × B10.Q F1 mice are susceptible to *Clostridioides difficile* infection. **(A)** Experimental setup of *C. difficile* infections. **(B)** Mean (± SEM) relative weight curves of mice gavaged with bacterial growth medium (n = 14) and infected mice (630: n = 19, 2K14: n = 16, VPI10463: n = 16). Asterisks denote statistically significant (**p-value = 0.0033) weight differences between experimental groups. **(C)** H&E stained 4 µm sections of colonic tissue depict erosive colitis on day 2 after infection with “summit lesions” (compare open arrowheads in I to edge of mucosal layer in IV), reduced numbers of vesicles (compare II and arrowheads in IV), thickened submucosal layer with inflammatory infiltrates, and capillary vessels in I (arrows). Scale bars represent 100 µm (I and III) and 50 µm (II and IV), respectively.

Data sets were analyzed for Gaussian distribution using Shapiro-Wilk tests. For comparison of two groups, t-tests and Mann-Whitney U-tests were performed, respectively. For comparisons of more than two groups, one-way ANOVA with *post hoc*-tests (Dunn’s multiple comparison; Tukey’s multiple comparison) were performed (**Figures 1B** and **5B**). Kaplan-Meier-Estimator was used to show differences in incidences of experimental arthritis performing log rank (Mantel-Cox), Breslow (generalized Wilcoxon), and Tarone-Ware-Tests. Correlations were calculated according to Spearman (non-parametric) or Pearson (parametric test). Analyses were performed using GraphPad Prism (Version 5, GraphPad Software, La Jolla, CA 92037 USA) or SPSS (Version 22, IBM, Armonk, NY, USA), respectively. P values smaller than 0.05 were considered statistically significant.

## Results

### DBA/1J × B10.Q F1 Mice Are Susceptible to Infection With *Clostridioides difficile*

The first experiment served to find out if our most susceptible mouse strain for collagen induced arthritis (CIA) would develop symptoms of clostridia induced diarrhea. To that extent, DBA1 x B10.Q F1 mice were pretreated for 3 days with a cocktail of antibiotics followed by a 2 days wash-out period. A single intraperitoneal injection of clindamycin on day 6 completed the disruption of the physiological microbiome before mice were challenged with 10^5^ cfu *C. difficile via* gavage on day 7 (**Figure 1A**). In order to compare three *C. difficile* strains that differ with respect to toxin production for their capacity to induce diarrhea, 19 mice were infected with the strain 630 (low toxin secretion), and 16 each with the strains 2K14 (medium toxin secretion) and VPI 10463 (very high toxin secretion), respectively (21, 28). An additional 14 mice received the antibiotics pretreatment followed by gavage with bacterial growth medium only.

After oral gavage, mice were scored daily for weight loss. As shown in **Figure 1B**, the antibiotic pretreatment led to a moderate weight loss in most mice and persisted for another 2 days after gavage with bacterial growth medium or strains 630 and 2K14, respectively. However, these treatment groups recovered quickly and within another 6 days surpassed their original body weight. In contrast, mice infected with the VPI 10463 strain lost a mean 5.4% of their body weight and were only back to a mean of 99% on experimental day 15. A one-way ANOVA performed for the weights on day 9 yielded a significant difference for VPI 10463 infected mice compared to all other groups. Note that all infected groups displayed comparable antibody titers against toxin A, while the least pathogenic strain 630 provoked the highest titers against toxin B (**Supplemental Figure 1**). To further explore if severe weight loss after VPI 10463 infection was associated with gut pathology, three mice were sacrificed on day 2 after infection and were screened for histological changes. Comparison to healthy colonic samples showed signs of severe erosive colitis (**Figure 1C**).

### Infection With *Clostridioides difficile* VPI 10463 Significantly Reduced the Incidence of Subsequent Collagen Induced Arthritis

**Figure 2:**
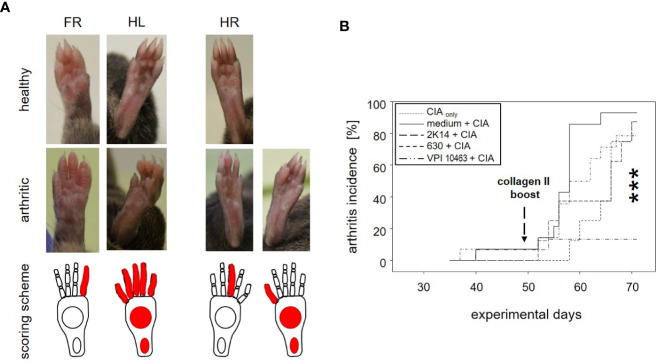
Infection with *Clostridioides difficile* significantly reduced incidence of collagen-induced arthritis in DBA/1J × B10.Q mice. **(A)** Macroscopic pictures of healthy (upper row) and arthritic paws (middle row) and corresponding scoring schemes (lower row) are shown. Middle row from left to right: an arthritis score of 1 (one inflamed digit), 15 (five inflamed digits, inflamed tarsal and metatarsal joints), 1 (one inflamed digit), and 11 (one inflamed digit, inflamed tarsal and metatarsal joints) respectively. **(B)** inverted Kaplan-Meier plot shows incidences of collagen-induced arthritis after mice were boosted on experimental day 49. Asterisks denote an extremely significant difference between the groups at the various time points as calculated *via* log rank (Mantel-Cox) test. The resulting p-value was 0.000363.

To assess the impact of *C. difficile* infection on the development of subsequent arthritis, we provoked CIA in F1 mice that were either treatment naïve (CIA_only_) or had previously been infected with either VPI 10463, 2K14, or 630 strains, respectively. To be able to differentiate any effects resulting from the initial antibiotic treatment, we also provoked CIA in animals that had been gavaged with bacterial growth medium, only (medium + CIA). After collagen II immunization, all animals were scored macroscopically by counting inflamed carpal, metacarpal, tarsal, and metatarsal joints as well as inflamed digits of front and hind paws. Examples are depicted in **Figures 2A, B** summarizes our results. For the medium group, incidence of arthritis rose the sharpest, shortly after the collagen type II boost on experimental day 49. At endpoint, 13 out of 14 mice (93%) had at some point during the observation period suffered from arthritis. Likewise, between 78.6 and 87.5% of the CIA_only_ mice and those pre-infected with 2K14 or 630 strains developed arthritis. In contrast, only 2 out of 15 mice (13%) previously infected with the VPI 10463 strain developed inflamed joints. A log rank (Mantel-Cox) test revealed extremely significant differences between the incidences of the various groups. In summary these results indicate that an initial antibiotics treatment accelerated subsequent arthritis while infection with the most aggressive VPI 10463 strain of *C. difficile* significantly attenuated this development. Of note, all animals immunized and boosted with collagen type II developed comparable antibody titers against bovine and murine collagen type II, confirming successful immunization and the capacity to mount a humoral immune response (**Supplemental Figure 2**).

### *Clostridioides difficile* Infections Were Paralleled by an Accelerated Reversal Towards the Original Microbiome After Antibiotics Treatment

**Figure 3:**
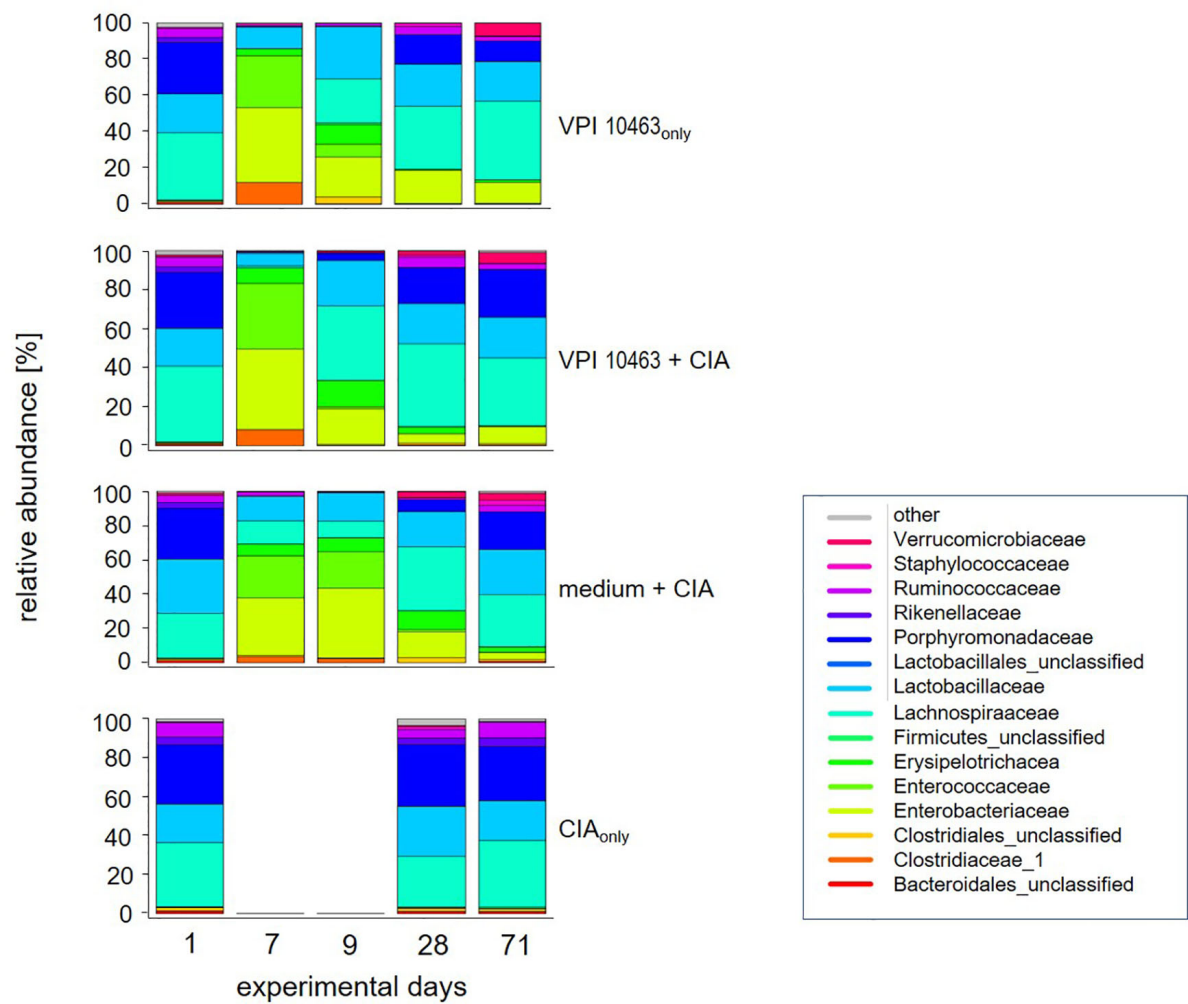
VPI 10463 infection accelerates the reversal towards the original microbiome after antibiotics treatment. Stacked bar plots show relative abundancies of bacterial species in stool samples on experimental days 1, 7, 9, 28 and endpoint for the various experimental groups.

The previous results led us to investigate whether the differential onset and incidence of arthritis after VPI 10463 infection or gavage with growth medium only could be linked to specific gut microbiomes. We therefore analyzed the microbial composition of stool samples from these experimental groups (medium+CIA and VPI 10463+CIA) and compared them to stool samples from mice that had suffered from either VPI 10463 infection or CIA induction, only. In detail, fresh stool pellets were collected at five time points throughout the experiments (**Figure 1A**). From these, DNA was extracted, the microbial 16S rRNA amplified and the sequencing data analyzed for relative abundancies. Before the onset of any antibiotic treatment on experimental day 1, all groups displayed a comparable microbial composition which is dominated by Porphyromonadaceae, Lactobacillaceae, Lachnospiraceae, and to minor abundancies of Ruminococcaceae and Rikenellaceae (**Figure 3**). Of note, this composition remained stable for the CIA_only_ group and indicated that arthritis induction was not paralleled by changes to the gut microbiome. All other groups received antibiotic treatment and thereafter, on experimental day 7 displayed a shift towards Enterobacteriaceae, Enterococcaceae, and Clostridiaceae. Following gavage with *C. difficile* or bacterial growth medium on experimental day 7, a reversal towards the original microbiome set in with increased abundancies of Lachnospiraceae and Lactobacillaceae. Interestingly, challenge with *C. difficile* seemed to accelerate the reversal towards the original microbiome whereas the group gavaged with bacterial growth medium only hosted Enterococcaceae and lacked Ruminococcaceae for an extended period. This trend was still visible on experimental day 28—the day of collagen II immunization—when Porphyromonadaceae and Ruminococcaceae showed increased abundancies in the VPI 10463 infected but not yet in the medium group. Of note, Verrucomicrobiaceae appeared at the end of the observation period and only in those groups that had been treated with antibiotics. In summary, comparing the various treatment groups for relative abundancies of their stool microbiota did not reveal any arthritis specific microbiomes yet indicated that the presence of *C. difficile* infections were paralleled by an accelerated reversal towards the original microbiome after antibiotics treatment.

### VPI 10463 Infection Following the Onset of Collagen-Induced Arthritis Lacked Therapeutic Potential

**Figure 4:**
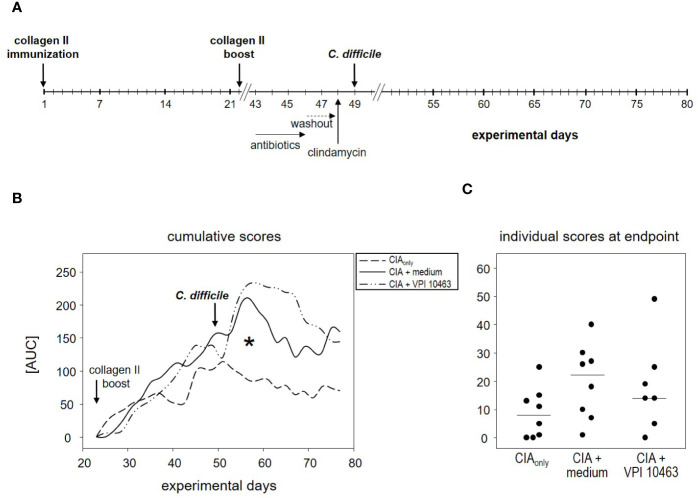
VPI 10463 infection following the onset of collagen-induced arthritis (CIA) lacked therapeutic potential. **(A)** experimental scheme with CIA induction preceding *Clostridioides difficile* infection. **(B)** Cumulative arthritis scores presented as area under the curve (AUC), from experimental day 22 until endpoint. The significant difference on day 57 was calculated *via* one-way ANOVA and resulted in a *p-value of 0.0270. **(C)** Individual arthritis scores at endpoint.

The finding that infection with the VPI 10463 strain reduced the incidence of subsequent arthritis (**Figure 2B**) led us to investigate the therapeutic potential of *C. difficile* infections. We therefore swapped our experimental procedures and provoked CIA first and infected the animals with VPI 10463 only after the onset of arthritis (**Figure 4A**). On day 43, all of the CIA_only_ mice and 6/8 within the CIA+VPI 10463 group had developed arthritis. Another one within the CIA+VPI 10463 group developed arthritis on day 45 while yet another one stayed completely free until the end of the observation period. Importantly, there was no resolution of arthritis following VPI 10463 infection. When looking at the cumulative arthritis scores, there was a sharp increase in the severity for the CIA+VPI 10463 and the CIA+medium groups following day 51 (**Figure 4B**). This increase resulted in a significantly different cumulative score among the three experimental groups on day 57. However at endpoint, all groups were again comparable with respect to their arthritis scores (**Figure 4C**). In summary, we did not observe any direct therapeutic effect of VPI 10463 infection on existing CIA. Instead, it seemed as if the treatment with the mix of antibiotics led to a transient increase in arthritis.

### Eradication of VPI 10463 and Subsequent Fecal Microbiota Transplantation Obliterated Any Attenuating Effect

**Figure 5:**
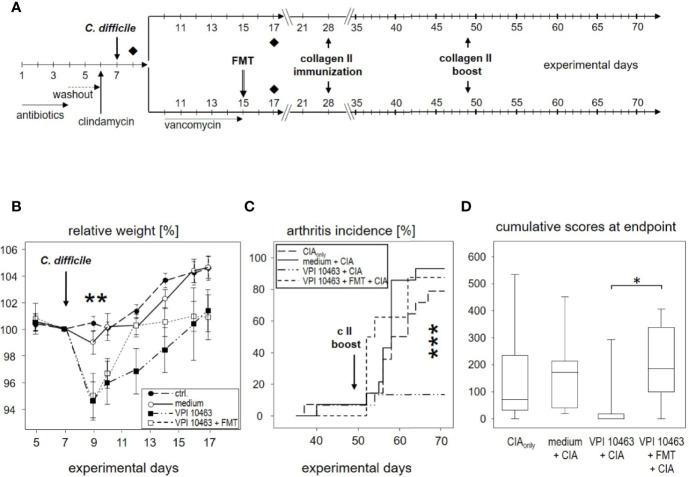
Eradication of VPI 10463 and subsequent fecal microbiota transplantation obliterated any attenuating effect. **(A)** experimental scheme with *Clostridioides difficile* infection, subsequent eradication, and fecal microbiota transplantation (FMT). **(B)** Mean (± SEM) relative weight curves of untreated controls (n = 8), mice gavaged with bacterial growth medium (n = 8), infected (VPI 10463: n = 8) and infected, eradicated, and fecal microbiota transplanted mice (VPI 10463 + FMT: n = 8). Asterisks denote significant weight differences between the experimental groups (p = 0.0057). **(C)** inverted Kaplan-Meier plot shows incidences of collagen-induced arthritis (CIA) with an extremely significant difference between the groups as calculated *via* log rank (Mantel-Cox) test (p-value = 0.000269). **(D)** Box-plots present cumulative arthritis scores at endpoint. A Kruskal-Wallis test resulted in a p-value of 0.0194.

We next asked whether the attenuating effect of VPI 10463 infection on subsequent arthritis (**Figure 2B**) was compatible with clearance of live *C. difficile* and reconstituting mice with normal flora. To address this question, we again infected mice *via* oral gavage with VPI 10463 and 2 days later eradicated *C. difficile by* administering vancomycin with the drinking water for 5 days. The day after, fecal microbiota from treatment naïve donor mice were transplanted and mice were allowed to recover for another 2 weeks, before CIA was provoked. Mice not receiving FMT did neither receive vancomycin (**Figure 5A**).

As can be seen in **Figure 5B**, vancomycin treatment on experimental day 9 helped VPI 10463 infected mice to quickly recover and regain their pre-infection weight on day 12. However, FMT treatment also obliterated any attenuating effect and led to arthritis incidences that were comparable to mice that had been gavaged with bacterial growth medium only or mice that were induced for CIA_only_ (**Figure 5C**). Likewise, cumulative arthritis scores at endpoint were comparable (**Figure 5D**). In summary, VPI 10463 infection attenuated subsequent CIA only in the absence of antibiotic eradication and FMT.

### Spontaneous Clearance of VPI 10463 Infection Led to Mesenteric T_reg_ and T_h2_ Polarization

**Figure 6:**
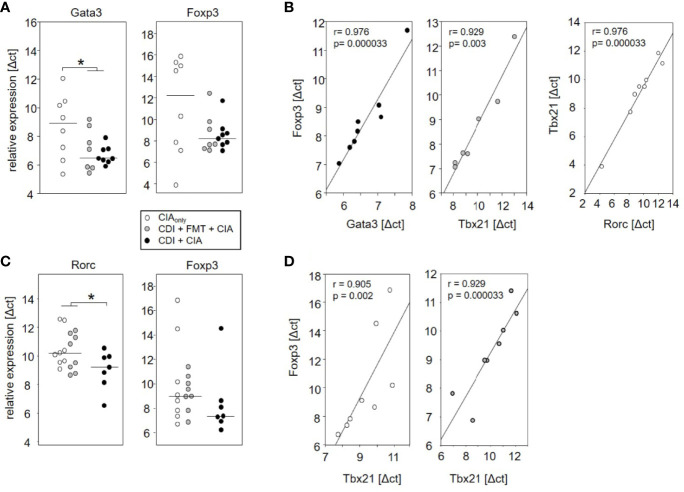
Spontaneous clearance of VPI 10463 infection led to mesenteric but not inguinal T_reg_ polarization. Dot plots show the differential expression of subset-specific transcription factors in the mesenteric **(A)** and inguinal **(C)** lymph nodes relative to Gapdh. The p-values resulting from t-test were 0.018 for A and 0.0325 for **(B)** Linear regression analyses indicate correlations among the expression of transcription factors **(B)** and between Gata3 expression and arthritis scores **(D)**. Calculations were performed *via* Spearman rank correlation analyses. Each dot represents an individual mouse.

The observation that VPI 10463 infection and its spontaneous clearance attenuated subsequent CIA whereas FMT treatment did not, prompted us to investigate whether there was differential imprinting on the immune responses. To that extent we collected mesenteric and inguinal lymph nodes at endpoint and compared their individual T helper cell profiles *via* subset-specific transcription factors. **Figure 6A** shows that VPI 10463 infection led to a long-lasting upregulation of Gata3 transcripts that was reminiscent of dominant T_h2_ responses. As this upregulation was paralleled by a trend towards increased Foxp3 expression, we were intrigued whether polarization existed among the T_regs_. Indeed, Foxp3 expression in the mesenteric lymph nodes of VPI 10463 infected mice that spontaneously cleared their infection correlated significantly with the respective Gata3 expression, indicting a non-inflammatory T_reg_-T_h2_ milieu. However, this was not true for the lymph nodes of FMT-treated animals. Here, Foxp3 expression correlated significantly with Tbx21 and Rorc expression, indicating a T_h1_ and T_h17_ polarization (**Figure 6B**). Of note, in the absence of VPI 10463 infection we did not observe any correlation of Foxp3 expression with either Gata3, Tbx21, or Rorc. Instead, Tbx21 correlated with Rorc expression. Other than that we did not observe any further significant correlations (**Table 1**).

**Table 1:** Correlations between transcription factor expression in mesenteric lymph nodes and arthritis score.

	Gata3 [Δct]	Tbx21 [Δct]	Rorc [Δct]	Foxp3 [Δct]	CIA [score]
CIA_only_					
Gata3 [Δct]	1				
Tbx21 [Δct]	0.357/0.385	1			
Rorc [Δct]	0.405/0.320	**0.976/0.00033**	1		
Foxp3 [Δct]	0.786/0.021	0.571/0.139	0.619/0.102	1	
CIA [score]	0.476/0.233	−0.024/0.955	0.024/0.955	0.238/0.570	1
CDI + FMT + CIA					
Gata3 [Δct]	1				
Tbx21 [Δct]	6.43/0.119	1			
Rorc [Δct]	0.464/0.294	0.821/0.023	1		
Foxp3 [Δct]	0.464/0.294	**0.929/0.003**	0.893/0.007	1	
CIA [score]	0.286/0.535	0.286/0.535	−0.179/0.702	0.000/1.000	1
CDI + CIA					
Gata3 [Δct]	1				
Tbx21 [Δct]	0.381/0.352	1			
Rorc [Δct]	0.810/0.015	0.357/0.385	1		
Foxp3 [Δct]	**0.976/0.000033**	0.357/0.385	0.762/0.028	1	
CIA [score]	−0.558/0.151	−0.140/0.742	−0.482/0.226	−0.647/0.083	1

Correlation coefficients/p-values resulting from Spearman correlation analyses. According to Bonferroni correction for multiple comparisons, p-values < 0.0033 were considered significant. Values in bold highlight statistically significant correlations.

When analyzing the inguinal as the draining lymph nodes of the hind extremities, we observed a small but significant increase in Rorc expression—indicating T_h17_ polarization—in those mice that seemed exempt from arthritis. These mice also showed a trend towards increased Foxp3 expression (**Figure 6C**). However, Foxp3 and Rorc expression did not correlate with each other ruling out T_reg_ polarization towards T_h17_ (**Table 2**). In the experimental groups that developed arthritis, Foxp3 expression correlated with Tbx21, indicating T_reg_ polarization towards T_h1_ (**Figure 6D**).

**Table 2:** Correlations between transcription factor expression in inguinal lymph nodes and arthritis score.

	Gata3 [Δct]	Tbx21 [Δct]	Rorc [Δct]	Foxp3 [Δct]	CIA [score]
CIA_only_					
Gata3 [Δct]	1				
Tbx21 [Δct]	0.595/0.120	1			
Rorc [Δct]	0.571/0.139	0.833/0.010	1		
Foxp3 [Δct]	0.810/0.015	**0.905/0.002**	0.690/0.058	1	
CIA [score]	0.762/0.028	0.643/0.086	0.429/0.289	0.738/0.037	1
CDI + FMT + CIA					
Gata3 [Δct]	1				
Tbx21 [Δct]	0.524/0.183	1			
Rorc [Δct]	0.429/0.289	0.857/0.007	1		
Foxp3 [Δct]	0.643/0.086	**0.929/0.001**	0.881/0.004	1	
CIA [score]	0.667/0.071	0.214/0.610	0.381/0.352	0.524/0.183	1
CDI + CIA					
Gata3 [Δct]	1				
Tbx21 [Δct]	0.143/0.760	1			
Rorc [Δct]	0.000/1.000	0.857/0.014	1		
Foxp3 [Δct]	0.607/0.148	0,286/0.535	0.464/0.294	1	
CIA [score]	−0.519/0.233	−0.445/0.317	−0.556/0.195	−0.334/0.465	1

Correlation coefficients/p-values resulting from Spearman correlation analyses. According to Bonferroni correction for multiple comparisons, p-values < 0.0033 were considered significant. Values in bold highlight statistically significant correlations.

In summary, the analyses of mesenteric and inguinal lymph nodes revealed different T cell polarizations. Spontaneous clearance of VPI 10463 infection involved T_h2_ polarization among the mesenteric while CIA involved T_h1_ polarization among inguinal T_regs_.

## Discussion

We here combined for the first time an infection with *C. difficile* and CIA in the mouse and demonstrated that the application of 10^5^ cfu of the *C. difficile* strain VPI 10463 led to diarrhea, significant weight loss and overt histological gut inflammation in DBA/1 x B10.Q F1 mice. The observation that 630 and 2K14 strains were less pathogenic awaits further elucidation, however we consider it possible that the differential capacities for spore formation and toxin production that result in a loss of barrier function in the intestines play a role (21, 29).

Our important result is the finding, that *C. difficile* infection significantly reduced incidence and severity of subsequent CIA. Indeed, Atarashi and colleagues previously demonstrated that colonization of mice by a defined mix of Clostridium strains not only provided an environment rich in transforming growth factor-beta but also affected Foxp3-positive T_reg_ numbers and function in the colon (30). In addition, small chain fatty acids like butyrate that are produced by the microbes were shown to induce the differentiation of T_regs_ (31). Even though we did not analyze specific metabolites, our RT-PCR analyses of the mesenteric lymph nodes indicated an increase in FoxP3 positive cells that was paralleled by an increase in Gata3, however only in those mice that were allowed to clear VPI10463 infections spontaneously. Whether indeed, small chain fatty acids directly induce T_regs_ or whether they promote *C. difficile* toxin A and B production and thus indirectly foster T_reg_ induction remains to be investigated (32). Vancomycin and FMT treatment resulted in an altogether different mesenteric milieu with Foxp3 expression correlating with Tbx21, being suggestive of T_reg_ polarizations towards T_h1_.

As plastic differentiation and stability changes of T_regs_ have frequently been shown to play vital roles in the progression of diseases, the exact nature of the Foxp3 expressing cells found in mesenteric and inguinal lymph nodes awaits further investigation (33, 34). Even though our RNA analyses leave room for speculation, they do not allow for a clear distinction between populations of either T_h2_ or T_h1_ cells and T_regs_ on the one hand and plasticity among Tregs on the other. Moreover, we have no proof yet that T_h2_ or T_reg_ spread from the gut to the joints and helped prevent arthritis however, the ameliorating effect of VPI 10463 infection on CIA is supportive of a gut-joint-axis. Likewise, we can only speculate that remnant and life VPI10463 are required as antibiotic eradication and transplantation of normal flora abolished any effect. The lack of a therapeutic effect after CIA induction may be explained by the timing and the inability of gut derived T_h2_ effector T cells to override the inguinal T_h1_ response. Future short term experiments will help to unravel in detail how VPI10463 infections attenuate subsequent arthritis.

Another recent publication suggested a protective gut-joint-axis by describing a delay and reduced severity of CIA following dextran sodium sulfate-induced colitis (35). Even though there were no specific gut pathogens involved, we would like to speculate that the loss of barrier function in the intestines—as also provoked by *C. difficile* toxin—may imprint a cytokine profile that protects the joints. As in our experiments antibodies against collagen type II were produced independent of *C. difficile* infection, we would like to dismiss an altogether immunosuppressive mechanism.

It needs to be pointed out though that Wu and colleagues reported the induction of T_h17_ development, production of disease-inducing autoantibodies and accelerated onset of arthritis after colonization of germfree K/BxN mice with Clostridiaceae (36). Even though germfree mice were shown to have an impaired immune tolerance (37), conflicting data seem to exist that call for a mechanistic clarification of the gut-joint-axis.

Our analysis of the gut microbiome over the course of the experiments allowed us to speculate that the onset of CIA is not necessarily dependent on a shift in the microbial composition however, the gut microbiome may impact on CIA development. Mice gavaged with bacterial growth medium only and the CIA_only_ groups differed merely by the treatment with antibiotics, yet the former showed even higher incidences and accelerated onset of arthritis. As opposed to the groups that were infected with *C. difficile*, the medium group displayed prolonged and high abundancies of Enterobacteriaceae and Enterococcaceae on experimental days 9, 28, and 71 (see **Figure 3**) and indeed, *E. coli* is one of the “usual suspects” involved in the pathogenesis of autoimmunity (38). Likewise, *Proteus mirabilis* has recently been described as the main microbial culprit in the causation of RA with the mechanistic link between the bacterium and human disease implying molecular mimicry (39). Our data therefore support the notion that alterations to the microbial composition are not mandatory for arthritis induction yet may foster its development.

The idea that antibiotic treatment predisposes for autoimmunity was recently brought forward by higher incidence and severity of CIA in mice treated with Clindamycin but not Vancomycin (40). Likewise, Maeda et al. demonstrated that dysbiosis in arthritis-prone SKG mice contributed to joint inflammation *via* activation of autoreactive T cells in the intestine. They claimed that the intestine is a site for initial T cell activation and concluded that the gut microbiota may activate innate intestinal immune cells that in turn stimulate T cells to enhance autoreactivity to the joints (41).

In summary, our results demonstrate that *C. difficile* induced an inflammation of the gut that protected from subsequent arthritis development. However, there are limitations to our study regarding the underlying mechanism. We cannot differentiate whether it is the microbial changes that contribute to arthritis protection or whether immune cell mobilization and/or polarization towards gut inflammation prevented the development of articular disease. It will therefore be of outmost importance to investigate in future experiments the distribution of mucosal and systemic T_h17_ and T_reg_ cells, the cytokine response in the serum and more detailed microbiome analyses during CIA development. However, the prospect of a protective benefit resulting from *C. difficile* infections or some byproduct thereof positively encourage further experiments.

## Data Availability Statement

The raw data supporting the conclusions of this article will be made available by the authors, without undue reservation.

## Ethics Statement

The animal study was reviewed and approved by Landesamt für Landwirtschaft, Lebensmittelsicherheit und Fischerei Mecklenburg-Vorpommern, Thierfelderstraße 18, 18059 Rostock.

## Author Contributions

CJS, KW, BK, and BM-H contributed substantially to the conception and design of the work. CJS, KW, ME, JV, MM, JS, KK, and SK contributed to the acquisition, analysis, and interpretation of data. CJS, KW, and BM-H drafted the work and all co-authors revised it critically for important intellectual content and approved the final version for publication. The authors agree to be accountable for all aspects of the work in ensuring that questions related to the accuracy or integrity of any part of the work are appropriately investigated and resolved. All authors contributed to the article and approved the submitted version.

## Conflict of Interest

The authors declare that the research was conducted in the absence of any commercial or financial relationships that could be construed as a potential conflict of interest.

## Abbreviations

CIA, collagen-induced arthritis; RA, rheumatoid arthritis; CDI, C. difficile-infection; FMT, fecal microbial transplant; VPI 10463, C. difficile-strain; 2K14, C. difficile-strain; 630, C. difficile-strain.
