# Supplementary material for: Infection With Clostridioides difficile Attenuated Collagen-Induced Arthritis in Mice and Involved Mesenteric Treg and Th2 Polarization
Source: Front Immunol. 2020 Oct 30;11:571049. doi: 10.3389/fimmu.2020.571049 (PMC7662472; doi:10.3389/fimmu.2020.571049)
Supplement: Supplementary file 1 [file DataSheet_1.pdf]

# *Supplementary Material*

## 1 Supplementary Figures

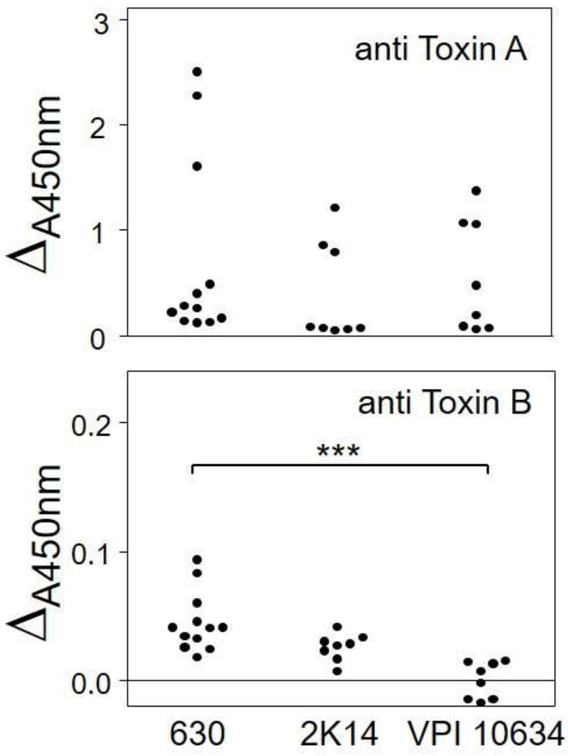


**Supplemental Figure 1.** Antibody titers against *C. difficile* Toxin A and B at the end of the experimental period. Each dot represents one mouse. Statistical analysis was performed using Kruskal-Wallis and posthoc tests (Dunn's multiple comparison test). Asterisks denote statistically significant differences resulting from Kruskal-Wallis. ***p<0,001.

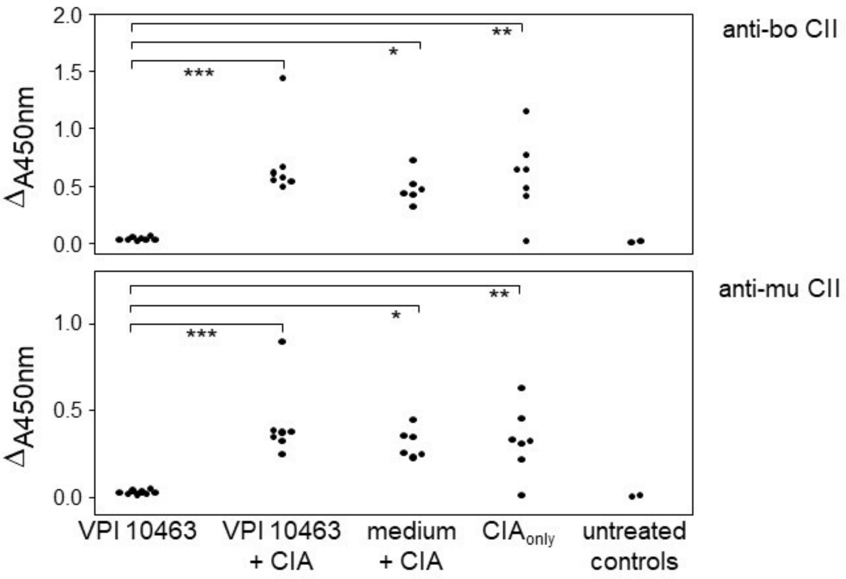


**Supplemental Figure 2.** Dot plots represent antibody titers against bovine and murine collagen type II. VPI 10463 (n=8), VPI 10463 + CIA = C. difficile infection + Collagen-induced arthritis (n=7), medium = gavage with bacterial growth medium + CIA (n=6), $CIA_{only}$ = Collagen-induced arthritis (n=6). Data are expressed as individual values. Statistical analysis was performed using one-way ANOVA with Tukey's Multiple Comparison Test *P<0.05, **P<0,01
